# DAFS: a data-adaptive flag method for RNA-sequencing data to differentiate genes with low and high expression

**DOI:** 10.1186/1471-2105-15-92

**Published:** 2014-03-31

**Authors:** Nysia I George, Ching-Wei Chang

**Affiliations:** 1 Division of Bioinformatics and Biostatistics, National Center for Toxicological Research, FDA, Jefferson, AR 72079, USA

**Keywords:** RNA-sequencing, Low expression, Data-adaptive, Flag, Mixture distribution

## Abstract

**Background:**

Next-generation sequencing (NGS) has advanced the application of high-throughput sequencing technologies in genetic and genomic variation analysis. Due to the large dynamic range of expression levels, RNA-seq is more prone to detect transcripts with low expression. It is clear that genes with no mapped reads are not expressed; however, there is ongoing debate about the level of abundance that constitutes biologically meaningful expression. To date, there is no consensus on the definition of low expression. Since random variation is high in regions with low expression and distributions of transcript expression are affected by numerous experimental factors, methods to differentiate low and high expressed data in a sample are critical to interpreting classes of abundance levels in RNA-seq data.

**Results:**

A data-adaptive approach was developed to estimate the lower bound of high expression for RNA-seq data. The Kolmgorov-Smirnov statistic and multivariate adaptive regression splines were used to determine the optimal cutoff value for separating transcripts with high and low expression. Results from the proposed method were compared to results obtained by estimating the theoretical cutoff of a fitted two-component mixture distribution. The robustness of the proposed method was demonstrated by analyzing different RNA-seq datasets that varied by sequencing depth, species, scale of measurement, and empirical density shape.

**Conclusions:**

The analysis of real and simulated data presented here illustrates the need to employ data-adaptive methodology in lieu of arbitrary cutoffs to distinguish low expressed RNA-seq data from high expression. Our results also present the drawbacks of characterizing the data by a two-component mixture distribution when classes of gene expression are not well separated. The ability to ascertain stably expressed RNA-seq data is essential in the filtering process of data analysis, and methodologies that consider the underlying data structure demonstrate superior performance in preserving most of the interpretable and meaningful data. The proposed algorithm for classifying low and high regions of transcript abundance promises wide-range application in the continuing development of RNA-seq analysis.

## Background

Transcriptome analysis is integral in understanding the role of genetic and genomic variants in disease progression, classification, and diagnosis. In recent years, next generation sequencing (NGS) technologies have catapulted transcriptome profiling to new heights by providing greater precision of cellular RNA content. As a result, RNA-seq is more sensitive to subtle changes in expression levels and typically includes many transcripts with low read count integers.

Researchers have taken interest in the low regions of a sample for several reasons, all with the primary intent of quantifying the level of expression that reflects important biological meaning. One of the major problems in RNA-seq data analysis is that there is no consensus on what is considered low expression. Low counts have been referenced as less than 10 reads when summed across treatments [1,2], less than 10 reads on average [3], less than 100 reads on average [4,5], and less than 300 reads [6]. Additionally, count-based pre-filtering methods have been adopted to exclude genes with minimal expression from differential testing. For example, Risso et al. [7] filtered out genes with an average count below 10. Robinson et al. [8] recommended removing genes that did not have at least 100 counts per million reads in at least two samples in the edgeR user’s guide. In the DESeq user’s guide, Anders [4] took a more aggressive approach and suggested removing up to 40% of genes that ranked lowest in regard to total count across all experimental samples. Thus, at present, whether low expressed transcripts are simply identified or deliberately omitted from analysis, methods of differentiating spurious RNA-seq data from meaningful information have not been explicitly defined. Furthermore, it is not clear whether it is justifiable to extrapolate the aforementioned cutoffs to other RNA-seq data.

A number of factors affect the distribution of read counts for a given study. Specifically, the manufacturer, library preparation and construction, alignment algorithm, gene length, sequencing depth, and experimental design all play a role in determining the number of reads that are mapped to a gene. Since each of these factors varies from study to study, it may be misleading to ignore properties of the sample distribution by applying an arbitrary cutoff to classify the low expressed region of a sample. To address the question of what should be considered the lower bound of functional gene expression, it is useful to consider the premise that genes can be categorized into a group of high expressed (HE), meaningful genes and a group of low expressed (LE), non-informative genes. In previous studies, the empirical distribution of a sample was used to identify classes of mRNA abundance levels [9]. This concept has also been utilized to differentiate functional expression states of microarray data [10-13]. In order to optimally separate expression classes, a priori knowledge of the expression distribution is useful. In many cases, the precise distribution of noise is unknown; however, characterizations of a global bimodal distribution and a normally distributed HE component have been reported in previous studies [10,14-16].

Hebenstreit et al. [16] studied the global distribution of RNA sequences in mice Th2 cells. Their work demonstrated that log2-transformed reads per kilobase per million (RPKM) could be separated into two classes of mRNA abundance. The researchers used the likelihood ratio test [17] to determine whether the data was best modeled as a mixture of *n* or *n* + 1 components, where 0 < *n <* 9. Mclust [18], an R package that uses an expectation-maximization (EM) algorithm, was used to compute maximum likelihood estimates of all parameters of the mixture distribution. For one-dimensional data, Mclust evaluates a fitted model with equal variance terms and a fitted model with unequal variance and selects the model with higher Bayesian Information Criterion (BIC) [19]. However, the use of mixture modeling to identify mixture components of RNA-seq data may not be appropriate for two reasons. First, it is difficult to capture the true number of mixture components. It is known that the EM algorithm performs well at estimating the parameters of a finite mixture model; however, when mixture distributions are unimodal and there is no clear separation between components, the EM algorithm commonly returns more components than what seems logical based on visual inspection of the data [20-22]. Second, when a two-component mixture model is forced to fit data that does appear to be bimodally distributed, the fitted model does not always approximate very well the observed empirical distribution.

Hebenstreit et al. [16] were able to characterize the mixture distribution of the LE and HE components and determine the peak of the LE region by using a Poisson distribution to estimate the proportion of undetected genes at each expression level. However, they did not discuss or provide an expression cutoff for separating the two overlapping regions. In this study, we propose DAFS, a data-adaptive method for identifying and subsequently flagging expressions in the LE region by estimating the lower bound of high expression in a given RNA-seq sample. In light of the drawbacks of the mixture modeling approach, DAFS was constructed without imposing a finite mixture model on the data. Several real RNA-seq datasets and simulated data are used to present our findings and demonstrate the robustness of our methodology.

## Results

**Figure 1:**
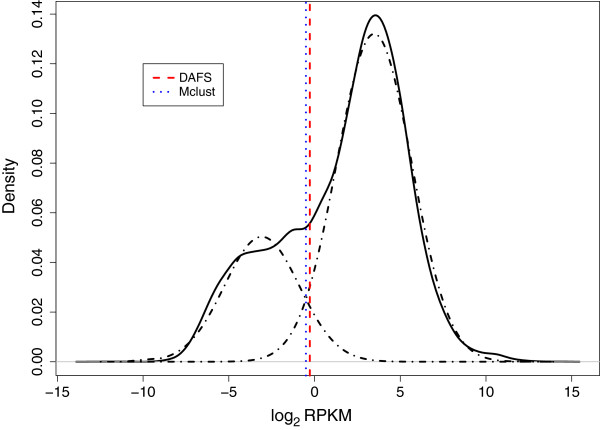
**DAFS and Mclust cutoff estimates for exon gene expression of Th2 mice cells.** The black solid lines are the original density of log2 RPKM data. The red dashed line and blue dotted lines present the estimated cutoffs for DAFS and Mclust, respectively. The fitted two-component mixture density is represented by the black dashed-dotted lines.

When the LE and HE regions of RNA-seq data are well separated (as they are in the distribution of exon gene expression resulting from averaged biological replicates of Th2 cells in Hebenstreit et al. [16], GSE28666), DAFS and Mclust separate the components at similar cutoffs (Figure 1). Cutoff values determined by DAFS and the theoretical intersection of the two mixture components obtained by Mclust were -0.3 and -0.5, respectively. In their study of Th2 cells, Hebenstreit et al. [16] identified a sample of known expressed and unexpressed genes in Th2 cells. Based on their classification of low and high expression, they mapped all the known expressed genes to the HE component and mapped most of the known unexpressed genes to the LE component (Supplementary Table S1 in [16]). We were able to reproduce their findings using the cutoff estimated by DAFS.

Analysis of bulk RNA positive controls generated from cultured HCT-116 cells [23] allowed for an additional assessment of the ability of DAFS to separate known expressed genes into one mixture component and known unexpressed genes into a separate mixture component. In their analysis of single-cell whole transcriptome amplification, the authors identified 40 known expressed and unexpressed genes in HCT-116 cells (Supplementary Table S2 in [23]). Fragments transformed by fragments per kilobase of exon per million mapped reads (FPKM) [24] were downloaded from the GEO database (GSE51254), and as specified in Wu et al. [23], were normalized according to median expression across all transcripts from a single cell and log2 transformed. The estimated DAFS cutoff of log2 median-adjusted FPKM data from RNA bulk was 0.78. At this cutoff all of the known expressed genes in the subset of 40 genes were mapped to the HE component and all of the known unexpressed genes were mapped to the LE component. The gene closest to the boundary of our cutoff for separating low and high expression was METTL3 (methyltransferase like 3) with a log2 median-adjusted FPKM value of 1.02. To assess the expression level of METTL3 in HCT-116 cells, we examined two microarray datasets from the GEO database (GSE32323, GSE11618) and confirmed that METTL3 is expressed in HCT-116 cells with low expression levels. Thus, to classify METTL3 in the HE component appears to be the appropriate decision based on biological evidence.

**Figure 2:**
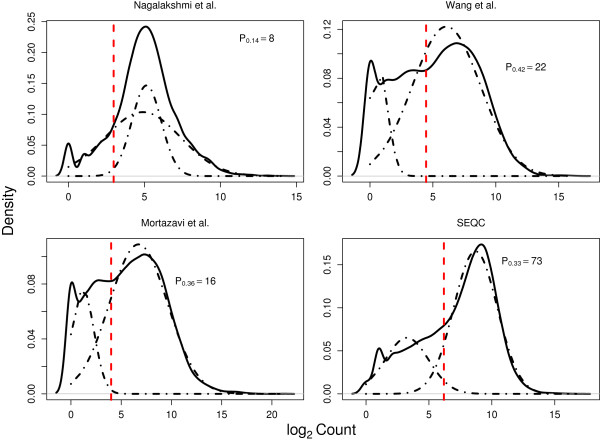
**DAFS cutoff estimates for four different empirical RNA-seq datasets.** The black solid lines are the original density of log2 raw count data. The red dashed lines present the estimated cutoffs. The fitted two-component mixture density is given by the black dashed-dotted lines.

To demonstrate the robustness of DAFS on distinct empirical distributions, four RNA-seq samples with unique expression profiles of log2-transformed raw counts were obtained from four different experimental studies. Similar to expression patterns observed in Hebenstreit et al. [16], every density exhibited a normally distributed component on the right (HE component) and a cluster of the remaining data on the left (LE component). However, each dataset differed in the degree of separation between the LE and HE component and in the distinct characterization of the LE region. Despite variation in the four different patterns of expression, DAFS performed consistently well at classifying data as LE and HE (Figure 2). In each sample, we were able to select a cutoff that clearly defined the HE component. From a visual perspective, the lower bound of the HE component, as identified by our approach, separated the data at a value that preserves as much of the normal HE component as possible. In other words, one would question normality of the HE component with a cutoff placed to the left of the estimated optimal quantile cutoff, *q*_*c*_, and one would forego valuable normally distributed data with a higher *q*_*c*_ value. Densities of the two-component mixture distribution obtained by Mclust are also presented in Figure 2. When the normal HE component is well characterized by Mclust, the theoretical cutoff and DAFS are close. The gap between estimated cutoffs widens as the model-data misfit increases.

DAFS estimates of *q*_*c*_ for each sample in Figure 2 demonstrate two additional important findings. Not only does the estimated quantile cutoff differ in each dataset, but the raw RNA-seq count corresponding to the computed quantile also differs. We identified quantile cutoff values of 0.14 (Nagalakshmi et al. [25]), 0.42 (Wang et al. [26]), 0.36 (Mortazavi et al. [27]), and 0.33 (SEQC). These percentiles mapped to raw counts of 8, 22, 16, and 73, respectively. This indicates the necessity of a data-adaptive feature. As evidenced by the results, it would not be appropriate to apply an arbitrary cutoff based on percentiles of the data nor on raw counts without consideration of the individual data structure of the sample.

Additionally, we analyzed two different RNA-seq datasets with technical replicates to assess the consistency of DAFS. Overall, DAFS demonstrated great consistency in estimating cutoffs from technical replicates. For SEQC, a *q*_*c*_ value of 0.33 was computed for each replicate of sample A. Values of *q*_*c*_ for replicates of sample B fluctuated between 0.24 and 0.25. In Hammer et al. [28], mRNA-seq in rats was measured 2 weeks and 2 months after L5 spinal nerve ligation (SNL). The study included two technical replicates for each treatment condition and also included a control for each time point. DAFS returned *q*_*c*_ values of 0.19 and 0.20 (control – 2 months); 0.20 and 0.16 (L5 SNL – 2 months); 0.23 and 0.24 (control – 2 weeks); 0.25 and 0.24 (L5 SNL – 2 weeks). Overall, DAFS showed small variability in estimating quantile cutoffs for technical replicated sequencing data. These results further demonstrate the impracticality of an arbitrary cutoff even within the same study. As evidenced by the data, it is reasonable to suspect that the separation of LE and HE genes is more homogeneous within replicates of the same experimental treatment. However, we may presume less agreement across biological replicates, treatments, and experiments.

**Figure 3:**
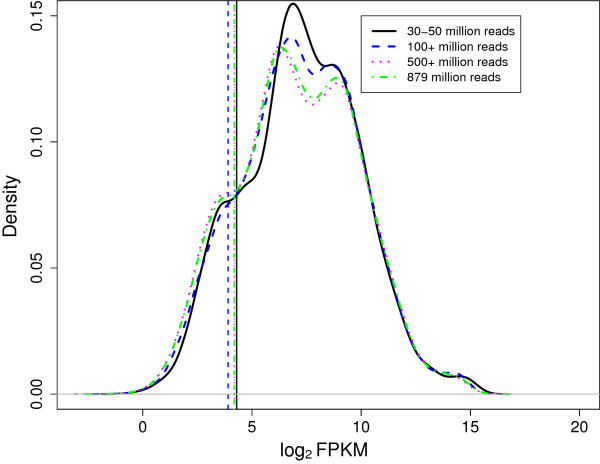
**DAFS cutoff estimates for different sequencing depths of cultured human B-cells.** Densities of log2 FPKM data for 30–50 million reads, 100+ million reads, 500+ million reads, and 879 million reads are presented by the solid black, dashed blue, and dotted magenta, and dash-dotted green line, respectively. The coordinated vertical lines present the DAFS cutoff estimate for each sequencing depth.

To explore further the spectrum of possible expression profiles, we measured the performance of DAFS on sequencing reads from cDNA fragments of cultured human B-cells (GSE12526) [29] in order to evaluate the functionality of DAFS on multi-modal data and data measured at various sequencing depths. As indicated in Toung et al. [29], we pooled all 20 unrelated samples obtained from the Center d’Etude du Polymorphisme Humain to create an 879 million read dataset. In addition, we randomly selected 1 sample and randomly pooled 3 and 12 samples to simulate sequencing depths of 30–50 million reads, 100+ million reads, and 500+ million reads, respectively. Densities of each distribution of log2 transformed FPKM data are provided in Figure 3. DAFS cutoffs for data composed of 30–50 million reads, 100+ million reads, 500+ million reads, and 879 million reads are 4.3, 3.9, 4.2, and 4.2, respectively. All are higher than the value of 2.3 that Toung et al. [29] used to classify low expression. Since the authors suggest that 500 million reads are necessary to accurately measure transcript expression, our findings indicate that cutoffs estimated by DAFS stabilize as the quality of the expression improves. Most importantly, although the log2 FPKM values are clearly multi-model, DAFS adapts to preserve most of the regions with high expression.

### Simulation

**Figure 4:**
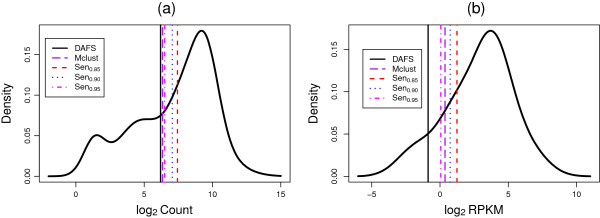
**Density plots with the average cutoff estimates.** The averaged cutoff estimates of DAFS, Mclust, Sen_0.85_, Sen_0.90_, and Sen_0.95_ for simulated **(a)** log2 raw counts and **(b)** log2 RPKM data were presented. The black lines are empirical densities of the data. The black vertical lines present the DAFS cutoff estimates. The purple long-dashed lines, red dashed lines, blue dotted lines, and magenta dot-dashed lines present cutoffs estimated by Mclust, 85% sensitivity, 90% sensitivity, and 95% sensitivity, respectively.

**Figure 5:**
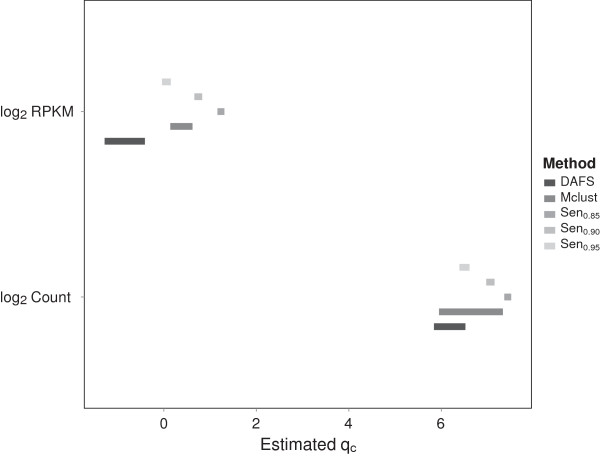
**Confidence intervals of cutoff estimates.** The confidence intervals were presented by the 2.5^th^ and 97.5^th^ quantile of the 500 simulated datasets.

To evaluate the performance of DAFS on simulated log2 count data and log2 RPKM data, sensitivity, specificity, positive predictive value (PPV), and negative predictive value (NPV) were evaluated for DAFS and Mclust (Table 1). The mean, 2.5% percentile, median, and 97.5% percentile of estimated cutoffs for DAFS, the point of intersection between theoretical distributions of the two-component normal mixture fitted by Mclust, and for empirical bounds associated with achieving sensitivity values of 0.85 (Sen_0.85_), 0.90 (Sen_0.90_), and 0.95 (Sen_0.95_) are reported in Table 2. For better visualization of the results, average cutoff estimates (Figure 4) and 95% confidence intervals for *q*_*c*_ (estimated by the 2.5^th^ and 97.5^th^ quantiles of cutoff estimates) (Figure 5) are provided for both simulation scenarios.

**Table 1:** Performance evaluation of DAFS and Mclust on simulated data

		**Mean ± SE**	
		**DAFS**	**Mclust**
Log2 Count	Sensitivity	0.9669 ± 0.0004	0.9529 ± 0.0015
	Specificity	0.8571 ± 0.0011	0.8683 ± 0.0021
	PPV	0.9235 ± 0.0005	0.9292 ± 0.0010
	NPV	0.9362 ± 0.0006	0.9173 ± 0.0022
Log2 RPKM	Sensitivity	0.9847 ± 0.0002	0.9292 ± 0.0005
	Specificity	0.7528 ± 0.0025	0.9462 ± 0.0007
	PPV	0.9701 ± 0.0003	0.9929 ± 0.0001
	NPV	0.8623 ± 0.0016	0.6253 ± 0.0015

**Table 2:** **Summary statistics of cutoff estimates from DAFS, Mclust, Sen**_**0.85**_**, Sen**_**0.90**_**, and Sen**_**0.95 **_**on simulated data**

		**Mean**	***P***_**25**_	**Median**	***P***_**97.5**_
	DAFS	6.1961	5.8407	6.1725	6.5163
	Mclust	6.3460	5.9477	6.0390	7.3297
Log2 Count	Sen_0.85_	7.4362	7.3597	7.4375	7.5078
	Sen_0.90_	7.0564	6.9687	7.0562	7.1524
	Sen_0.95_	6.5013	6.3909	6.5025	6.6131
	DAFS	-0.8736	-1.2729	-0.8023	-0.4078
	Mclust	0.3630	0.1361	0.3710	0.6187
Log2 RPKM	Sen_0.85_	1.2391	1.1612	1.2359	1.3143
	Sen_0.90_	0.7428	0.6551	0.7408	0.8341
	Sen_0.95_	0.0575	-0.0422	0.0598	0.1545

For log2 transformed count data, which presented a clear bimodal distribution, the estimated cutoffs from DAFS and Mclust were similar. Average sensitivity for both methods exceeded 95%. On average, the theoretical cutoff estimated by Mclust was closer to the 95% sensitivity estimate. Although Mclust shows an improvement in specificity and DAFS does a better job of optimizing the sensitivity and NPV, the differences in performance measures were small. Analysis of the 95% confidence intervals for cutoff values demonstrated that both DAFS and Mclust were not significantly different from estimates obtained from achieving 95% sensitivity.

In evaluating cutoffs for log2 transformed RPKM data, DAFS returned an average sensitivity estimate that exceeded 98%. However, Mclust estimates fell between values obtained for achieving 90% and 95% sensitivity. Analysis of the 95% confidence intervals for cutoff values presented similar findings. The results indicated that DAFS captured a higher proportion of true high expression. As such, DAFS demonstrated superior performance in sensitivity and NPV, whereas PPV was comparable between Mclust and DAFS. The low specificity of DAFS is explained by the proportion of low expression. Since the low expressed region represented roughly 10% of the total sample size, a small number of misclassified observations will decrease specificity rapidly.

Based on the two different scenarios, the results suggest that DAFS is comparable to Mclust at maximizing sensitivity when the mixture components are well separated; however, DAFS is superior at providing a cutoff that maximizes sensitivity when the LE and HE components are not distinguishable. The simulation results clearly demonstrate how DAFS adapts to different data patterns. The pattern of log2 RPKM data is relatively closer to one normal distribution than the log2 raw counts. Therefore, DAFS adjusts and retains as many genes as possible when analyzing log2 RPKM data. Consequently, relative to the results obtained from simulating log2 raw counts, sensitivity and PPV are increased but specificity and NPV are decreased.

## Discussion

This study was motivated by the need to provide a data-adaptive algorithm for separating RNA-seq data with low and high expression, particularly when the distributions of expression abundance are not distinctly separated. In order to compute the optimal cutoff between low and high expression, our method relied heavily on the assumption that high-expressed data are normally distributed [16]. An advantage of using the Kolmogorov-Smirnov distance as a measure of agreement is that the method is easily modifiable if a different distributional assumption is required to characterize high expression. For any continuous distribution, the K-S statistic tests the null hypothesis *H*_0_ : *F*(*x*) = *F*_0_(*x*), for all *x* versus the alternative *H*_1_ : *F*(*x*) ≠ *F*_0_(*x*), for some *x*. Thus, the reference distribution *F*_0_(*x*), e.g. normal, lognormal, Student’s t, etc., is completely specifiable by the researcher. To our advantage, DAFS performed consistently well when the HE component departed from normality (as evidenced in log2 RPKM and FPKM data).

Careful consideration should be taken with regard to the number of observations used to model the distribution of K-S statistics. The benefit of adding more data to characterize the underlying density should be balanced with the disadvantage of modeling added noise. If the predictor space is segmented too finely, then it is possible for multiple percentiles to map to the same K-S statistic. Multiple many-to-one mappings would make it difficult for the MARS algorithm to differentiate true variation from random noise. In the present study, incrementing *p* by step sizes of 0.01 or 0.005 provided a good balance between parsimonious and over-saturated input. Nevertheless, the increment selection is a variable in the proposed methodology that must be investigated by the researcher.

We were not remiss to consider the presence of more than two components of expression abundance. Since the Gaussian mixture model can well approximate the shape of any density [30], the number of Gaussian mixture components was estimated for multiple datasets. When Mclust was allowed to estimate the number of Gaussian mixture components, the algorithm often returned multiple mixture components. A similar finding was presented in the supplemental material presented of Hebenstreit et al. [16]. In their analysis of real datasets, values of AIC and BIC indicated that the data would be better fit by a *k* > 2 component Gaussian mixture. In our own analysis, many of the identified components were not separated enough to be heterogeneous populations. We employed a number of methods/packages to merge Gaussian mixture components (e.g. *fpc*[22], *pdfcluster*[31], *REBMIX*[32]) with no success. Nearly every method struggled by either distinguishing no separation or overly characterizing the distribution of abundance levels. The latter scenario was more pronounced in LE regions, where it seemed apparent that a number of mixture components were necessary to estimate non-Gaussian density regions. It became clear that variability in the expression data made it difficult to ascertain whether homogeneous sub-mixtures could be interpreted as a single component.

In our web search of the literature, the question of a cutoff for low expression in RNA-seq was frequently asked. Some questions were motivated by a desire to quantify what is considered expressed in RNA-seq. Others were motivated by a need to classify the level of measurement that could be trusted in assessing the significance of differential expression in low-expressed regions, particularly since research shows that the precision of RNA-seq data analysis improves as genes are more highly expressed. Whether transcripts with low expression are simply flagged or removed prior to testing via independent filtering, the work presented here provides a data-driven methodology for separating RNA-seq expression into meaningful components. Providing an accurate separation of RNA-seq data that is not based on *ad hoc* techniques or methodology that may be prone to model-data misfit will facilitate interpreting the quality of sequencing reads and lead to improved power for differential detection of high expressed, reliable data.

## Conclusions

In this study, we presented a method for classifying transcripts with low and high expression that promises wide-range application. The robustness of DAFS was demonstrated by applying DAFS to a number of RNA-seq data samples (real data examples and simulations) that varied by sequencing depth, species, normalization, and density shape.

## Methods

### Data

Several datasets were used to the test the performance of our methodology.

### SEQC

Part III of the Microarray Quality Control was an FDA-led, collaborate work to evaluate the technical performance of sequencing quality control (SEQC). In SEQC, reference RNA samples included the universal human reference RNA (UHHR) from Agilent/Stratagene (sample A) and the human brain total RNA (HBRR) from Life Technologies Corp. (sample B). External spike-ins were added to both samples for purposes of testing validation and assessing accuracy. In order to minimize sources of technical variance in our study, we restricted our analysis to gene-level Illumina RNA-seq data generated from one library preparation, processed on one lane, and obtained from one sequencing site. In total, our analysis included eight replicates of sample A and eight replicates of sample B.

### ReCount datasets

To demonstrate the ability of DAFS to handle various empirical data structures, four additional datasets were downloaded from the ReCount webpage [33]. RNA-seq from transcriptome analysis of yeast, humans, rats, and adult mice were obtained from Nagalakshmi et al. [25], Wang et al. [26], Hammer et al. [28], and Mortazavi et al. [27], respectively. All four studies were sequenced using Illumina/Solexa sequencing technology. Sequence reads were summarized into gene counts using Ensembl 61 annotation.

### The data-adaptive flag method for RNA-sequencing data (DAFS)

The algorithm for carrying out DAFS on a single sample is comprised of several steps. Since zero reads present no information, the first step consists of removing the zero counts from analysis. After removing the zero counts and transforming the data to log2 scale, the Kolmogorov-Smirnov statistic is applied to segmented quantiles of the empirical distribution. Finally, the multivariate adaptive regression splines function is fit to the distribution of Kolmogorov-Smirnov statistics and spline knots are used to determine the optimal cutoff for high expression.

### The Kolmogorov-smirnov statistic

The Kolmogorov-Smirnov (K-S) statistic is a quantitative measure of the maximum distance between the empirical distribution function of a sample and the cumulative distribution function of a reference distribution. In the present study, we rely on the K-S statistic to determine a cutoff for data attributed to the HE region by assessing agreement between the empirical distribution of assumed HE genes and the reference distribution. RNA-seq data after logarithmic transformation is approximately normally distributed. Thus, from a practical standpoint, we assumed a normal reference distribution for HE genes and used log2-transformed data to compute the optimal cutoff.

Suppose *X*_1_, …, *X*_*n*_ are i.i.d. random variables from an unknown mixture distribution function. Let *Y*_*p*_ = {*X*_*i*_|*X*_*i*_ > *X*_(*p*)_, *where p is the p*^*th*^ *quantile of X*}. The Kolmogorov distance can then be defined as:

$$D=\sqrt{n\left(1-p\right)}\underset{y}{\text{max}}\left|F_{p}\left(y\right)-F\left(y\right)\right|,$$

where *F*_*p*_ is the empirical distribution of the sample, *Y*_*p*_, and *F* is the cumulative distribution function of the assumed normal reference distribution. To best differentiate between LE and HE genes, the percentile cutoffs ranging from *p*_0_ to 0.50 in increments of either 0.01 or 0.005 (dependent upon the data structure) was considered. For each sample, *p*_0_ is the proportion of data aggregated at the minimum expression level e.g. the percentage of 0 values when analyzing log2-transformed raw RNA-seq. The decision to exclude minimum expression levels was motivated by a desire to eliminate a proportion of the noise produced by extreme low counts. The stopping value is set at 0.50 to allow for at least half the data to be used in analysis. This seems fitting to adequately describe the normal mixture component of HE genes. For each *i*^*th*^ observation of {*p*}, a corresponding K-S statistic is computed, denoted by *D*_*i*,_, *i* = 1, 2, …, length of {*p*}.

**Figure 6:**
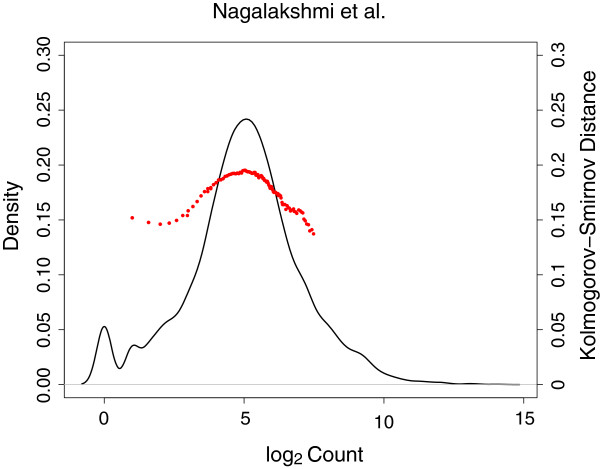
**A profile of the Kolmogorov-Smirnov distances for a sample taken from Nagalakshmi et al.** The black solid line is the original density of log2 raw count data. The red dots present the distribution of K-S statistics from *p*_0_ to *p* = 0.90.

A profile of the Kolmogorov-Smirnov distances for a sample taken from Nagalakshmi et al. [25] is presented in Figure 6. For plotting purposes, the distribution of K-S statistics from *p*_0_ to *p* = 0.90 is presented. Global analysis of the profile of K-S statistics demonstrates that the minimum K-S distance, i.e. the minimum distance between the observed distribution and theoretical normal distribution, occurs where the large normal distribution is clearly distinguishable. Empirical densities of other RNA-seq datasets (not shown) showed similar K-S profiles across percentiles of the data.

### Cutoff classification

The distribution of K-S statistics, {*D*}, across percentiles of the sample is used to classify the differentiation between data with low and high expression. Ideally, the data should be separated where {*D*} achieves a local minimum. To estimate the slope change points in Kolmogorov distances, multivariate adaptive regression splines (MARS) [34] was used to model *f* : *p* → *D*.

#### 1) The MARS algorithm

MARS is an adaptive nonparametric regression algorithm that uses piece-wise linear basis functions to model non-linear relationships. The predictor space is partitioned by knots into subregions to fit splines that distinguish segments of differing slopes. Let {*D*} and {*p*} be as previously defined, then *D* ⊂ *R*^*p*^ can be described by a general regression model,

$$D_{i}=f\left(p_{i}\right)+\varepsilon ,$$

where *ε* denotes residual error. The MARS algorithm approximates *f* using recursive partitioning [35,36] to expand *f* into linear combinations of basis functions and is denoted by a model of the form:

$$\hat{f}\left(x\right)=\sum_{m=1}^{M}\alpha_{m}B_{m}\left(x\right),$$

where M is the number of spline basis functions and *B*_*m*_ and *α*_*m*_ are the *m*^*th*^ spline function and its regression coefficient, respectively. Points that signify a break in the sample space and describe distinct linear splines are of key importance. These knots pinpoint a change in the model function, which indicate critical points of marked changed in K-S distances.

#### 2) Optimal quantile cutoff, *q*_*c*_

The MARS algorithm uses a forward and backward stepwise selection algorithm to automatically determine the basis functions and set of knots or breakpoints to partition the predictor space. For a continuous variable, MARS defines the sub-regions under which spline coefficients *α*_*m*_ are stable through the use of linear basis functions of the form:

$${\left(x-t\right)}_{+}=\left\{\begin{array}{ll} x-t, & \text{if}\;x>t,\\ 0, & \text{otherwise}, \end{array}\right)$$

and

$${\left(t-x\right)}_{+}=\left\{\begin{array}{ll} t-x, & \text{if}\;x<t,\\ 0, & \text{otherwise}, \end{array}\right)$$

where *t* is a spline knot selected from observed values of *x*. Friedman [34] controls the span size (distance between knots) by evaluating the minimum size needed to describe a smoothed function. An over-complicated model is built up in the forward stepwise procedure. Basis functions (and knots) are systematically deleted in the backward pruning process until an optimal set of knots is selected that describes the underlying data structure without being overly influenced by random fluctuations of the data. The best-fitting MARS model is chosen as the sub-model that minimizes prediction error measured via the generalized cross-validation (GCV) score [37]:

$$\text{GCV}\left(M\right)=\frac{\sum_{i=1}^{N}{\left[y_{i}-f_{M}\left(x_{i}\right)\right]}^{2}}{N{\left[1-\left(C\left(M\right)/N\right)\right]}^{2}},$$

where *N* is the number of observations and *C*(*M*) is the cost complexity function of *M* basis functions [38]. This criterion also provides an optimal balance between bias and variance.

The final MARS model includes the minimum number of necessary knots to capture the true model. Thus, the selection of knots is used to identify critical points along the range of K-S statistics. The optimal quantile cutoff, *q*_*c*_, is determined by the quantile *p* such that *f* has a local minimum. In the presence of multiple local minima, *q*_*c*_ = min{*q*_*c*_} i.e. the left-most point indicating a decreasing-to-increasing change in slope. The MARS algorithm was performed using the ‘earth’ package in R.

R script to generate quantile cutoffs for a sample is provided in Additional file 1: Supplementary Materials.

### Theoretical cutoff of Two-component normal mixture model

Let *x*_1_, …, *x*_*n*_ be independent, identically distributed random observations with density

$$f\left(x|\theta \right)=\pi_{1}\varphi_{1}\left(x|\mu_{1},\sigma_{1}^{2}\right)+\pi_{2}\varphi_{2}\left(x|\mu_{2},\sigma_{2}^{2}\right)$$

where *π*_*k*_ > 0 ∀ *k* = 1, 2 is the mixing weight of the *k*^*th*^ Gaussian distribution, $\sum_{k=1}^{2}\pi_{k}=1$, and $\varphi_{k}\left(x|\mu_{k},\sigma_{k}^{2}\right)$

is the density of the *k*^*th*^ Gaussian mixture with mean *μ*_*k*_ and variance $\sigma_{k}^{2}.$ The point of intersection between $\varphi_{1}\left(x|\mu_{1},\sigma_{1}^{2}\right)$ and $\varphi_{2}\left(x|\mu_{2},\sigma_{2}^{2}\right)$ specifying where

$$\varphi_{1}\left(x|\mu_{1},\sigma_{1}^{2}\right)=\varphi_{2}\left(x|\mu_{2},\sigma_{2}^{2}\right)$$

is given by:

$$x_{\text{evar}}=\frac{2\sigma^{2}\ln\left(\frac{\pi_{1}}{\pi_{2}}\right)+\left(\mu_{2}-\mu_{1}\right)\left(\mu_{1}+\mu_{2}\right)}{2\left(\mu_{2}-\mu_{1}\right)}$$

under an equal variance mixture model with *σ* = *σ*_1_ = *σ*_2_ and by:

$$x_{u\;\text{var}}=\frac{\mu_{2}\left(v^{2}-1\right)-v^{2}\left(\mu_{2}-\mu_{1}\right)\pm \sqrt{v^{2}{\left(\mu_{2}-\mu_{1}\right)}^{2}+2\sigma_{2}^{2}\left(v^{2}-1\right)\ln\left(v*\frac{\pi_{1}}{\pi_{2}}\right)}}{\left(v^{2}-1\right)},$$

where *v* = *σ*_2_/*σ*_1_ when the variance terms are unequal. *x*_evar_ or *x*_uvar_ were used to determine the theoretical cutoff between expression abundance classes when two-component mixture modeling was used to fit the distribution of LE and HE regions.

### Simulated data

Two simulation studies based on distributions of log2 transformed raw counts and log2 transformed RPKM data were proposed to demonstrate the ability of DAFS to differentiate LE/HE regions. For the first scenario, Mclust was used to fit the distribution of log2 raw counts of sample A from the SEQC study with no restrictions placed on the number of mixture components. The proportion, mean, and variance parameter estimates of the fitted 5 component mixture model were ${\hat{\pi }}_{1}=\left(0.08,0.28,0.17,0.26,0.21\right)$, ${\hat{\mu }}_{1}=\left(1.33,4.68,7.86,9.50,9.32\right)$, and ${\hat{\sigma }}_{1}^{2}=\left(0.62,2.83,1.17,0.90,3.53\right)$. RPKM data from Wang et al. [39] and made available through GEO Accession viewer was used to derive the second simulation. Parameters estimates of the proportion, mean, and variance of log2 RPKM data were ${\hat{\pi }}_{2}=\left(0.11,0.24,0.25,0.31,0.09\right)$, ${\hat{\mu }}_{2}=\left(‒1.80,1.30,3.40,4.20,6.60\right)$, and ${\hat{\sigma }}_{2}^{2}=\left(1.80,1.80,1.80,1.80,1.80\right)$.

In the simulation of log2 raw counts (scenario 1), the first two components were treated as LE. In scenario 2, only the first component of log2 RPKM data was treated as LE. To evaluate DAFS’s performance with competing methodology, results of DAFS were compared to cutoffs determined by fixing values of sensitivity and by cutoffs determined by the point of intersection between the two theoretical distributions of a fitted two-component Mclust model. Several methods for selecting an optimal cutoff have been proposed based on specific underlying objectives e.g. cost-benefit analysis or diagnostic test accuracy measures (sensitivity/specificity, predictive values, and diagnostic likelihood ratios). In this study, since the objective is to retain as many of the HE genes as possible, the decision criterion is based on setting sensitivity. Empirical cutoffs were computed for sensitivity values set to 0.85 (Sen_0.85_), 0.90 (Sen_0.90_), and 0.95 (Sen_0.95_). The total number of genes was fixed at 5,000 and the simulation was repeated 500 times.

### Availability of supporting data

R script to generate quantile cutoffs for a sample is provided in Additional file 1: Supplementary Materials.

## Abbreviations

DAFS: Data-Adaptive Flag Method for RNA-Sequencing Data; NGS: Next-generation sequencing; RNA-seq: whole transcriptome sequencing; HE: High expressed; LE: Low expressed; RPKM: Log2-transformed reads per kilobase per million; FPKM: Fragments per kilobase per exon model per million mapped reads; EM: Expectation-maximization; SNL: Spinal nerve ligation; PPV: Positive predictive value; NPV: Negative predictive value; K-S: Kolmogorov-Smirnov; MARS: Multivariate adaptive regression splines; GCV: Generalized cross-validation.

## Competing interests

The authors declare that they have no competing interests.

## Authors’ contributions

NIG performed the real data analyses and wrote the first draft of the manuscript. CWC conceived the study and performed the simulation study. Both authors participated in discussions related to analysis and interpretation and have read and approved the final manuscript.

## Supplementary Material

Additional file 1 Supplementary materials. Click here for file
