# Supplementary material for: DAFS: a data-adaptive flag method for RNA-sequencing data to differentiate genes with low and high expression
Source: BMC Bioinformatics. 2014 Mar 31;15:92. doi: 10.1186/1471-2105-15-92 (PMC4098771; doi:10.1186/1471-2105-15-92)
Supplement: Additional file 1 — Supplementary materials. [file 1471-2105-15-92-S1.doc]

Additional file 1: **SUPPLEMENTARY MATERIALS**

Note: This supplementary file is organized in two sections:

1. Data Loading and preprocessing
2. Main Method function

**1. Data Loading and Preprocessing**

#required libraries

#all packages can be found at R (<http://www.r-project.org/>)

library(mclust) ###used to estimate the parameters of reference distribution

library(earth) ###used to apply MARS algorithm

#Note: the data can be downloaded from ReCount

#(http://bowtie-bio.sourceforge.net/recount/)

#create infile and outfile directories

#Ifdir is the path for saved data

#Ofdir is the path for output result

#For example, Ifdir <- "C:/My Documents/RNASeq_Datasets/"

data <- read.delim(paste(Ifdir,dataset,"_count_table.txt",sep=""),sep="\t",header=T)

temp <- data

data <- temp[,-1]

rownames(data) <- temp[,1]

#determine which rows have all 0 counts

out <- apply(data,1,function(x) all(x==0))

if(length(out[out=="TRUE"])>0) data <- data[-which(out=="TRUE"),]

**2. Main Method function**

#the function requires two inputs: data and name

#data is the after preprocessing count data

#name is the file name of output result

qcut <- function(data,name) {

#set vector for cutoff values

cutv <- rep(0,0)

for (i in 1:ncol(data)) {

#specify array and remove 0 counts

xx <- data[,i]

xx <- xx[-which(xx==0)]

#take log2 of data

log2xx <- log2(xx)

dlog2 <- data.frame(LogC=log2xx)

#vector to store Kolmogorov Smirnov distance statistics

vv <- rep(0,0)

#select start point

start <- length(log2xx[log2xx==min(log2xx)])/length(log2xx)

#set sequence

s <- seq(round(start,2),0.5,by=0.005)

#loop through cuts of the data to determine targeted K-S statistic

for(q in s) {

#select data greater than a quantile and run Mclust on that data to determine theoretical distribution

d <- log2xx[which(log2xx>quantile(log2xx,q,na.rm=T))]

out <- Mclust(d,G=1)

ks <- ks.test(d,"pnorm",out$parameter$mean,

out$parameter$variance$sigmasq)

vv <- c(vv,ks$statistic)

}

#determine first left-most local minima

out <- earth(s,vv,thresh=0.005)

#save suggested cut

cutv <- c(cutv,min(out$cuts[out$cuts>0]))

}

names(cutv) <- colnames(data)

#send results to outfile

write.csv(cutv, paste(Ofdir, "QCutoff_", name, ".csv",sep=""),

row.names=F)

}
